# Structural and biochemical evaluation of bisubstrate inhibitors of protein arginine N-methyltransferases PRMT1 and CARM1 (PRMT4)

**DOI:** 10.1042/BCJ20190826

**Published:** 2020-02-27

**Authors:** Emma A. Gunnell, Alaa Al-Noori, Usama Muhsen, Clare C. Davies, James Dowden, Ingrid Dreveny

**Affiliations:** 1 School of Chemistry, University of Nottingham, Nottingham NG7 2RD, U.K.; 2 Centre for Biomolecular Sciences, School of Pharmacy, University of Nottingham, Nottingham NG7 2RD, U.K.; 3 College of Medical and Dental Sciences, University of Birmingham, Edgbaston, Birmingham B15 2TT, U.K.

**Keywords:** active site, crystallography, inhibition, PRMT, protein arginine methylation, small molecules

## Abstract

Attenuating the function of protein arginine methyltransferases (PRMTs) is an objective for the investigation and treatment of several diseases including cardiovascular disease and cancer. Bisubstrate inhibitors that simultaneously target binding sites for arginine substrate and the cofactor (*S-*adenosylmethionine (SAM)) have potential utility, but structural information on their binding is required for their development. Evaluation of bisubstrate inhibitors featuring an isosteric guanidine replacement with two prominent enzymes PRMT1 and CARM1 (PRMT4) by isothermal titration calorimetry (ITC), activity assays and crystallography are reported. Key findings are that 2-aminopyridine is a viable replacement for guanidine, providing an inhibitor that binds more strongly to CARM1 than PRMT1. Moreover, a residue around the active site that differs between CARM1 (Asn-265) and PRMT1 (Tyr-160) is identified that affects the side chain conformation of the catalytically important neighbouring glutamate in the crystal structures. Mutagenesis data supports its contribution to the difference in binding observed for this inhibitor. Structures of CARM1 in complex with a range of seven inhibitors reveal the binding modes and show that inhibitors with an amino acid terminus adopt a single conformation whereas the electron density for equivalent amine-bearing inhibitors is consistent with preferential binding in two conformations. These findings inform the molecular basis of CARM1 ligand binding and identify differences between CARM1 and PRMT1 that can inform drug discovery efforts.

## Introduction

**Figure 1.**
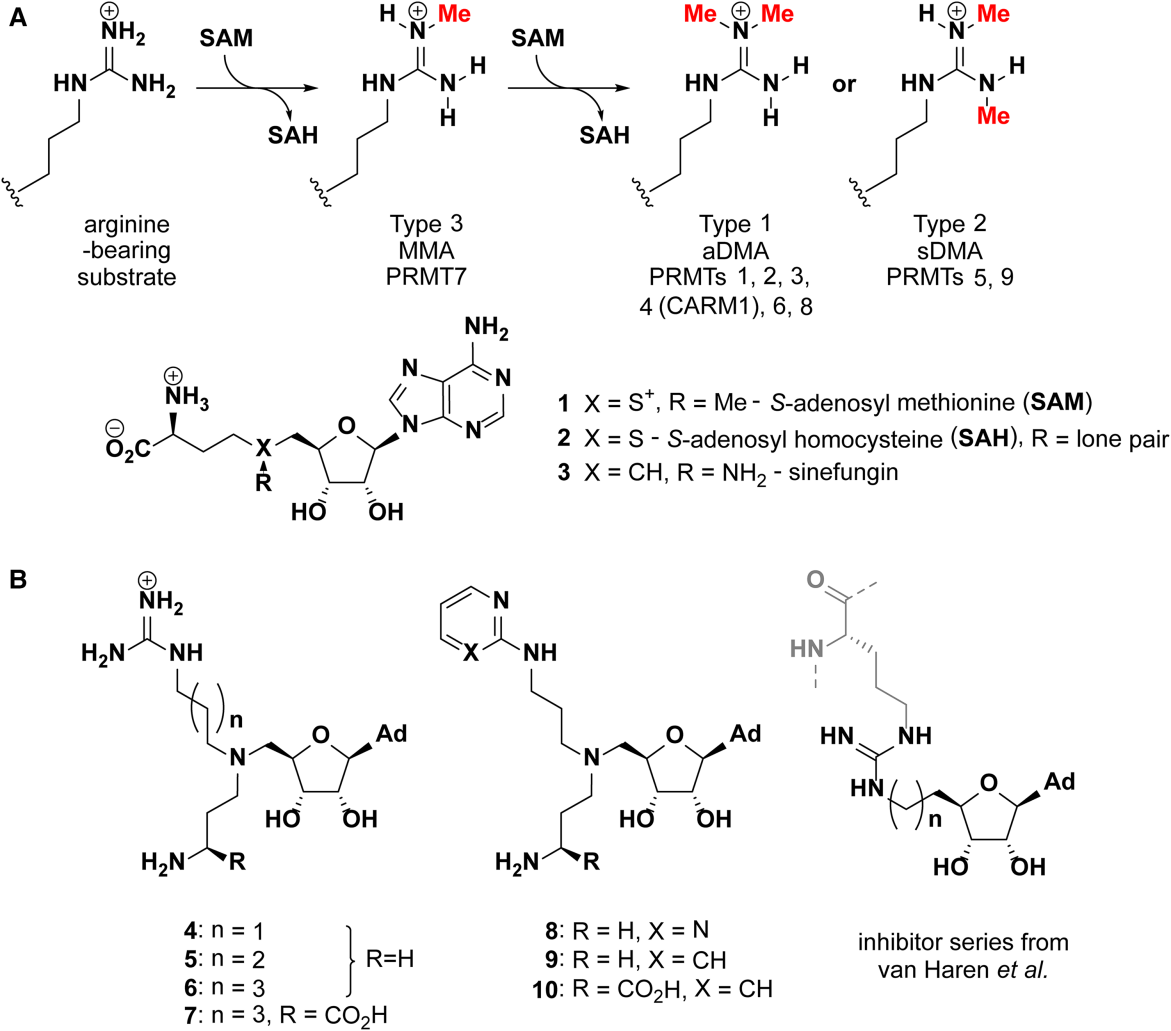
PRMT classification and inhibitor structures. (**A**) Arginine methylation patterns deposited by PRMTs. The PRMTs are classified as type 1, 2 or 3 according to whether they generate monomethylarginine (MMA), symmetric dimethyl arginine (sDMA) or asymmetric dimethylarginine (aDMA); (**B**) Structures of bisubstrate azo-SAM inhibitors from the present study (**4**–**10**) and those reported previously by van Haren et al. [28,33].

Protein arginine methyltransferases (PRMTs, E.C. 2.1.1.125) catalyse the transfer of methyl groups from *S-*adenosylmethionine (SAM) to arginine residues of target proteins, giving a pattern of nitrogen methylations dictated by enzyme type (Figure 1A). Hydrogen bonding N-H moieties are replaced by hydrophobic N-methyl groups to effect protein–protein interactions as a result. Co-activator associated arginine methyltransferase (CARM1, or PRMT4) targets substrates that contain proline-rich sequences rather, than the GGRGG sequences favoured by PRMT1. PRMTs are involved in the regulation of numerous cellular processes including chromatin function [1–8], RNA processing [9,10], and DNA-damage response [11–13]. Co-activation of gene transcription is effected by methylation of histone arginines: H4R3 by PRMT1; or H3R17 and H3R26 by CARM1, for example. Meanwhile, methylation of RNA binding proteins is involved in the regulation of protein translation. An increasing body of evidence links PRMT dysregulation to disease processes, including asthma, kidney and cardiovascular disease and cancer [14–17], underlining the need for potent, well-characterised PRMT inhibitors as molecular probes and potential therapeutic agents. CARM1 is overexpressed in breast [18], prostate [19], lung [20], liver [21], and colorectal cancer [22]. CARM1 inhibition has been shown to slow tumour growth in a multiple myeloma xenograft model [23], inhibit liver cancer cell proliferation [21] and impair AML initiation and proliferation [24]. PRMT1 on the other hand catalyses the majority of methylation events in the cell and is overexpressed in breast cancers, prostate cancers, and leukemia [15].

PRMT inhibitors reported to date can be broadly categorised into SAM analogues (including the pan-methyltransferase inhibitors sinefungin (**3**) and *S-*adenosyl homocysteine (**2**), (Figure 1A)), substrate peptide analogues [25–28], and structures optimised from high throughput screening hits [29–32]. Promising CARM1 inhibitors include EPZ022302/EZM2302 (a 6 nM CARM1 inhibitor with anti-tumour activity *in vivo*) [23] and TP-064 (IC_50_ < 10 nM against CARM1 and >100-fold selective over other methyltransferases) [32]. In both cases, ternary crystal structures with CARM1-SAH showed the inhibitor to occupy the substrate-binding pocket with kinetic data supporting noncompetitive inhibition with respect to both SAM and peptide substrate [23,32]. Alternatively, inhibitors may occupy both the SAM and arginine binding sites (Figure 1B) [28,33–35]. A number of such inhibitors that target CARM1 feature amino adenosine linked to modified cytosine moieties that occupy the SAM and arginine binding site, respectively [36]. The most potent of these inhibitors displayed an IC_50_ of 1.5 µM against CARM1, and was 100-fold less potent against PRMT1. Design of selective inhibitors is challenging however, since the residues involved in SAM and substrate arginine binding are largely conserved in the nine PRMTs encoded in the human genome, particularly between type I family members [37]. In line with recent advances [38], the development and structural investigation of rationally designed bisubstrate inhibitors remains a desirable route to identify individual residue variations that may contribute to selective inhibition of CARM1 over PRMT1 for example.

Bisubstrate inhibitors featuring guanidinium and aza-adenosylmethionine moieties joined by alkyl linkers were previously reported. Longer linkers appeared to confer a degree of selectivity between CARM1 and PRMT1, which was rationalised based on docking studies [34]. Van Haren et al. have also explored this approach by 5′-hydroxyl group of adenosine with a methylguanidinium group, which resulted in a compound with 100-fold selectivity for CARM1 over PRMT1 [33]. Extending the guanidinium group to a CARM1 substrate peptide achieved a greater degree of selectivity for CARM1 over PRMT1 (IC_50_ values of 0.09 and 25.5 µM, respectively for the most selective compound) [28]. Whilst promising in their apparent selectivity, a key drawback of such peptide-fused mimics is their potential susceptibility to proteolysis, which limits their utility in cellular assays and *in vivo* applications.

Novel non-peptidic bisubstrate inhibitors and crystal structures of their complexes with CARM1 are reported here. Isothermal titration calorimetry and activity assays allowed measurement of affinity and inhibition of both PRMT1 and CARM1. Data herein shows that replacement of the guanidinium group with an aminopyridine or aminopyrimidine enhances the inhibitors’ affinity for CARM1 but not PRMT1. The influence of a specific active site residue on the orientation of the catalytic glutamate and inhibitor binding was evaluated with CARM1 N265Y mutant protein; crystal structures revealed that this mutation affects the conformation of key residues at the substrate-binding site.

## Experimental procedures

### Constructs, protein expression and purification

The catalytic domain of human CARM1, residues 135 to 479 (CARM1_135–479_; isoform 3, UniProt accession code Q86X55) was cloned into the vector pMALX(E) (a modified pMAL-c2x vector, kindly provided by Lars Pederson [39]) using restriction sites *NotI* and *HindIII* with the addition of a C-terminal His-tag and a TEV cleavage site directly upstream of the CARM1 sequence. The final sequence expressed was MBP-AALAAAQTNAAAENLYFQ-CARM1_135–479_-HHHHHH. CARM1_135–479_ was expressed in BL21-CodonPlus (DE3)-RIL cells (Agilent) at 20°C after induction with 0.4 mM IPTG. After 20 h, cells were harvested by centrifugation, resuspended in 50 mM Tris, 300 mM NaCl, 20 mM imidazole, 5% v/v glycerol at a pH of 7.5 (buffer A), lysed by sonication, and the lysate clarified by centrifugation. CARM1_135–479_ was purified by nickel affinity chromatography (5 ml HiTrap chelating HP column, GE Healthcare) using buffer A with a gradient elution of 20 to 500 mM imidazole over 20 column volumes. The protein was concentrated to ∼1 mg/ml and cleaved with TEV protease to remove the MBP tag. The cleaved protein was then separated from TEV protease and the MBP tag by gel filtration (HiLoad Superdex S200 16/60 PG, GE Healthcare), fractions containing the protein concentrated and 1 mg/ml aliquots either used directly or flash frozen and stored at −80°C until further use. Approximately 0.2 mg CARM1 catalytic domain was obtained per litre of bacterial culture.

Human PRMT1, residues 22 to 361 (PRMT1_22–361_; isoform 1/splice variant 2, Uniprot accession code Q99873), was cloned into vector pET-26b(+) using restriction sites *NdeI* and *XhoI*. PRMT1_22–361_ was expressed and purified as outlined for CARM1, except that buffers were at pH 8.0 and the His-tagged protein obtained from nickel affinity chromatography was concentrated and loaded directly onto a gel filtration column. Approximately 2 mg of PRMT1 were obtained per litre of bacterial culture.

The sequence for histone peptide H4 residues 1 to 21 (herein referred to as H4_1–21_) and the sequence for histone peptide H3, residues 3–31 (herein referred to as H3_3–31_) were cloned into a modified pET-21a(+) vector containing an N-terminal GB tag and a C-terminal His-tag. H4_1–21_ and H3_3–31_ were expressed and purified as described above for PRMT1 except that the buffers had a lower NaCl concentration of 150 mM. Aliquots of the peptides were concentrated to 400 µM, flash frozen, and stored at −80°C until needed.

### Inhibitor compounds

Synthesis of **4** and **7** was described previously [34], **5**, **6**, **8**, **9** and **10** were prepared similarly and are described in the Supplementary Information.

### Crystallisation

Co-crystallisations of CARM1_135–479_ with inhibitors were carried out in 24-well plates using the sitting-drop vapour diffusion method. In the case of compound **5**, the CARM1 catalytic domain was concentrated to 1.7 mg/ml, followed by addition of a 500 µM aqueous stock solution of inhibitor such that the final protein concentration was 28 µM and the final ligand concentration was 167 µM. Co-crystallisations were performed by mixing 1 µl of the protein-inhibitor solution with 1 µl of a solution containing 0.1 M Bis-tris propane-Cl at a pH of 7.0, 0.02 M potassium phosphate and 22% (w/v) PEG 3350 in sitting drop trays. Trays were incubated at 20°C and crystals grew within one week. Co-crystallisations with the other inhibitors were carried out with similar ligand concentrations and temperatures, but the crystallisation conditions, and CARM1 concentrations varied as detailed in Supplementary Table S1. Crystals were cryoprotected in the well solution supplemented with 30% (v/v) glycerol and 20% (w/v) PEG 3350 prior to cryocooling in liquid nitrogen.

### Data collection, structure determination and refinement

Datasets were collected at the European Synchrotron Radiation Facility, Grenoble, France or Diamond Light Source, Harwell, Oxford using beamlines I02, ID30B and ID30A-1. Crystals belong to space group P2_1_2_1_2, with four monomers in the asymmetric unit. All CARM1 structures were solved by molecular replacement using Phaser MR [40]. The structure of CARM1 in complex with sinefungin (**3**) and an inhibitor (PDB code 2Y1X) [41] or subsequently CARM1 in the CARM1–**5** complex structure were used as search models in molecular replacements (with the ligands removed). Model building was carried out in COOT [42] and refinement was carried out with Phenix [43] and Refmac [44]. Ligand restraints were generated using eLBOW [45]. All structural images were generated using The PyMOL Molecular Graphics System, Version 1.8 Schrödinger, LLC. Parameters for data collection, structural refinement and validation are listed in Supplementary Table S2.

### Enzymatic assays

Methylation reactions contained PRMT1_22–361_ (160 nM), H4(1–21) (40 µM) and adenosyl-l-methionine, *S-*[methyl-^3^H] (PerkinElmer, NET155H250UC) at a final concentration of 0.23 µM, in phosphate buffered saline buffer at pH 7.3 in the presence of the different inhibitors. For IC_50_ curves, a minimum of 10 inhibitor concentrations were used, and experiments were carried out in duplicate. The reactions were initiated by addition of adenosyl-l-methionine, *S-*[methyl-^3^H] and incubated at 30°C after mixing. After 60 min, optimised for signal to noise ratio in the linear region, each reaction mixture was transferred by pipette onto a P81 phosphocellulose paper square (PerkinElmer) and immediately immersed in a stirred solution of trichloroacetic acid (10% w/v). The phosphocellulose squares were washed for 3 × 10 min, replacing the 10% trichloroacetic acid after each wash. A final wash was carried out with 95% ethanol, the squares removed and allowed to dry. Incorporation of tritium into the histone substrate was determined by liquid scintillation counting. For each inhibition series, a no-substrate control reaction was included, which contained water in place of the histone substrate. This data point was included in the data used to fit the curve by setting the *x*-value (log[I]) to 4.0. A no-inhibitor control was also included in the data, with the *x*-value (log[I]) set to −3.0. These control samples were also run in duplicate.

CARM1 methylation reactions were carried out using 0.23 µM adenosyl-l-methionine, *S-*[methyl-^3^H], 320 nM CARM1_135–479_ and 120 µM histone substrate (H3(3–31)). Since CARM1 automethylates, a no-enzyme control was used as the maximum inhibition sample (log[I] set to 4.0). Data were analysed in GraphPad Prism 7 and fit using non-linear regression using the ‘log(inhibitor) vs. response — variable slope (four parameters)’ function with no constraints. The concentrations of inhibitor solutions were determined using a NanoDrop 1000 v3.7, using the molar extinction coefficient at 260 nm for *S-*adenosyl homocysteine (ε = 15 400 M^−1^ cm^−1^). 2-alkylaminopyrimidines (**8**) and 2-alkylaminopyridines (**9** and **10**) absorb at 260 nm with extinction coefficients of <15 400 M^−1^ cm^−1^ [46,47]. The concentrations of **8**, **9** and **10** may therefore have been overestimated by up to two-fold with corresponding effect on the estimated *K*_d_ and IC_50_ values for these inhibitors.

### Isothermal titration calorimetry (ITC)

Titrations were carried out using a MicroCal PEAQ-ITC Isothermal Titration Calorimeter (Malvern) or MicroCal iTC200 (Malvern) in the case of the titration of **5** into PRMT1_22–361_. Data analysis was carried out using MicroCal PEAQ-ITC Analysis Software. Inhibitors were dissolved in gel filtration buffer to concentrations between 150 and 736 µM as suitable for ITC assays. An amount of 2.4 µl injections of inhibitor solutions into the protein solution at concentrations between 21 and 121 µM were performed, at 25°C. Control titrations were carried out by titrating the inhibitor solution into the buffer solution. The control heats of dilution were subtracted from binding experiments prior to curve fitting using a one-site binding model with fitted offset. The raw data and integrated fitted curves are shown in Supplementary Figures S2, S4, S6–S8 derived thermodynamic parameters are listed in Supplementary Tables S3, S4. Protein concentrations were determined by UV absorbance at 280 nm (with a NanoDrop 1000 v3.7) using extinction coefficients at 280 nm determined by the ExPASy ProtParam online tool. The protein concentrations calculated in this manner were shown to be in good agreement with the concentration determined by Bradford assay using a BSA standard. Inhibitor concentrations were determined as for activity assays. In two instances (**7** into PRMT1 and **10** into CARM1 N265Y), low c-value curves necessitated fixing the stoichiometry prior to curve-fitting.

## Results

### Inhibitors of both PRMT1 and CARM1 that bind in a bisubstrate manner

Putative bisubstrate inhibitors **4**, **5** and **6** containing amines and arginine-mimicking alkylguanidinium groups with chain lengths increasing from three to five methylenes respectively (Figure 1B) were assessed for their potential to bind and inhibit CARM1 and PRMT1. A radiometric methylation assay was used to assess the inhibitory strength of this series of inhibitors (Supplementary Figure S1) and resulting IC_50_ values are shown in Table 1, entries 1–3. Comparison of IC_50_ values for inhibitors **4**–**6** revealed a modest decrease in potency (2.3 to 17.7 µM) against CARM1_135–479_ as the length of the alkyl linker between the aminoadenosine and guanidinium groups is increased from 3 to 5 methylene units. No such trend was observed for PRMT1, with inhibitors displaying similar IC_50_ values for PRMT1_22–361_ (7.2–11.8 µM) as CARM1. Isothermal titration calorimetry was then used to determine the dissociation constants of this inhibitor series for PRMT1 and CARM1 (Table 1; Supplementary Figure S2; Supplementary Table S3). The resultant *K*_d_ values showed a similar pattern to the IC_50_ values, with all inhibitors binding with low micromolar affinity to both PRMT1 and CARM1 and again, CARM1 displayed a slight preference for a shorter alkyl linker length (Table 1). Inhibitor **7** (the amino acid analogue of **6**), was also evaluated and found to bind and inhibit both PRMT1 and CARM1 to a similar extent (Table 1; Supplementary Figure S2; Supplementary Table S3).

**Table 1:** *K*_d_ and IC_50_ values for series of inhibitors (4–10) and SAH against PRMT1 and CARM1 measured using ITC and enzyme activity assays, respectively 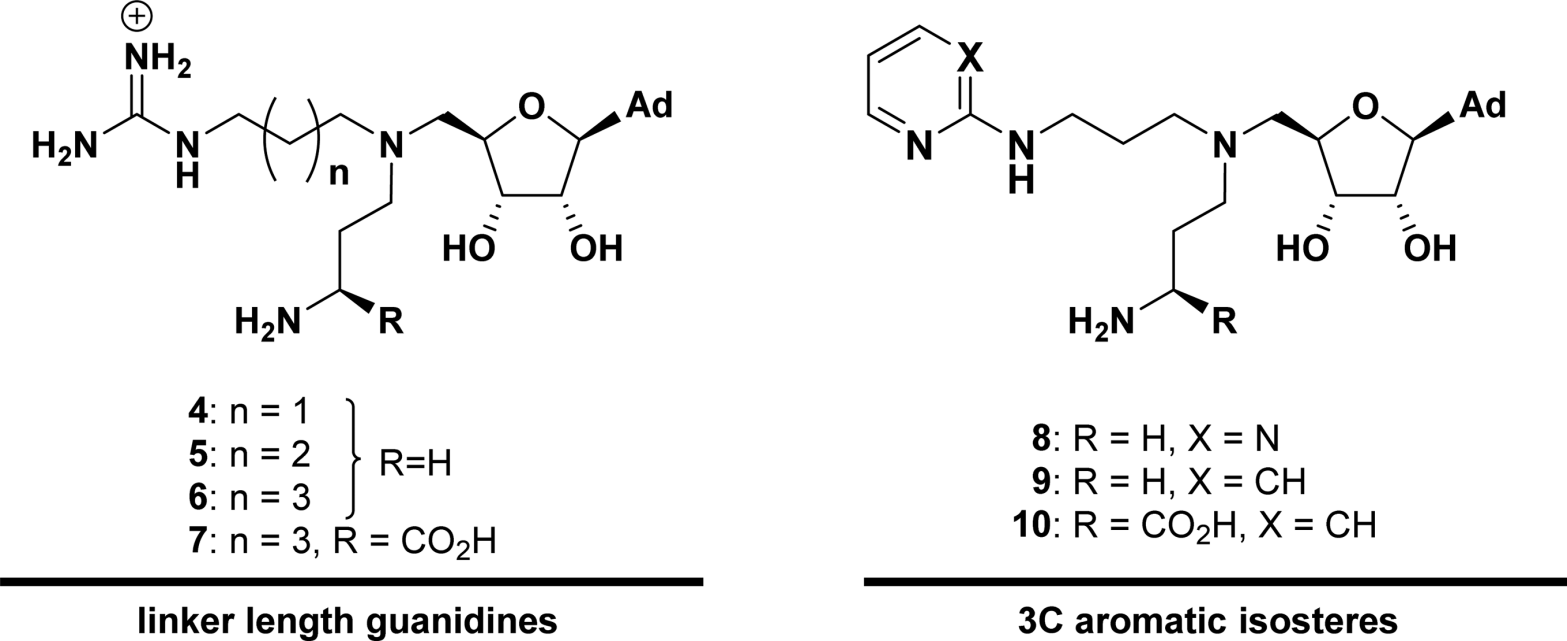

Inhibitor	*K*_d_ ± standard error (µM)		IC_50_ (95% confidence interval) (µM)	
	PRMT1	CARM1	PRMT1	CARM1
**4**	10.2 ± 0.996	3.32 ± 0.335	7.2 (3.5–15.0)	2.3 (1.8–3.0)
**5**	4.02 ± 0.453	7.18 ± 0.557	11.8 (9.0–15.5)	8.7 (6.9–10.8)
**6**	9.99 ± 1.89	9.80 ± 1.83	11.6 (4.9–27.2)	17.7 (13.5–23.2)
**7**	32.2 ± 3.53^1^	38.1 ± 9.61	22.0 (14.4–33.5)	24.1 (15.4–37.6)
**8**	10.2 ± 4.21	2.30 ± 0.162	22.0 (15.7–30.8)	0.7 (0.5–1.0)
**9**	15.3 ± 1.57	1.14 ± 0.411	11.1 (5.6–21.8)	0.3 (0.1–1.1)
**10**	43.7 ± 14.9^2^	1.11 ± 0.132	25.3 (17.3–37.0)	0.8 (0.6–1.0)
**SAH**	0.709 ± 0.057	0.775 ± 0.111	0.5 (0.2–1.0)	0.2 (0.1–0.4)

1 Stoichiometry fixed to 0.5.

2 This *K*_d_ is derived from a competition experiment with SAH as the *K*_d_ was not accessible through direct titration.

Co-crystallisation trials were carried out with the aim of determining the binding mode and whether the molecules bound as bisubstrate inhibitors. The catalytic core of CARM1 was crystallised in the presence of amine-guanidine inhibitors **4**, **5**, **6** and **7**, and the structures solved by molecular replacement. Crystals belonged to space group P 2_1_2_1_2 with two CARM1 dimers in the asymmetric unit, in line with CARM1 structures that have been previously reported [23,41,48]. Data collection and refinement statistics are shown in Supplementary Table S2.

**Figure 2.**
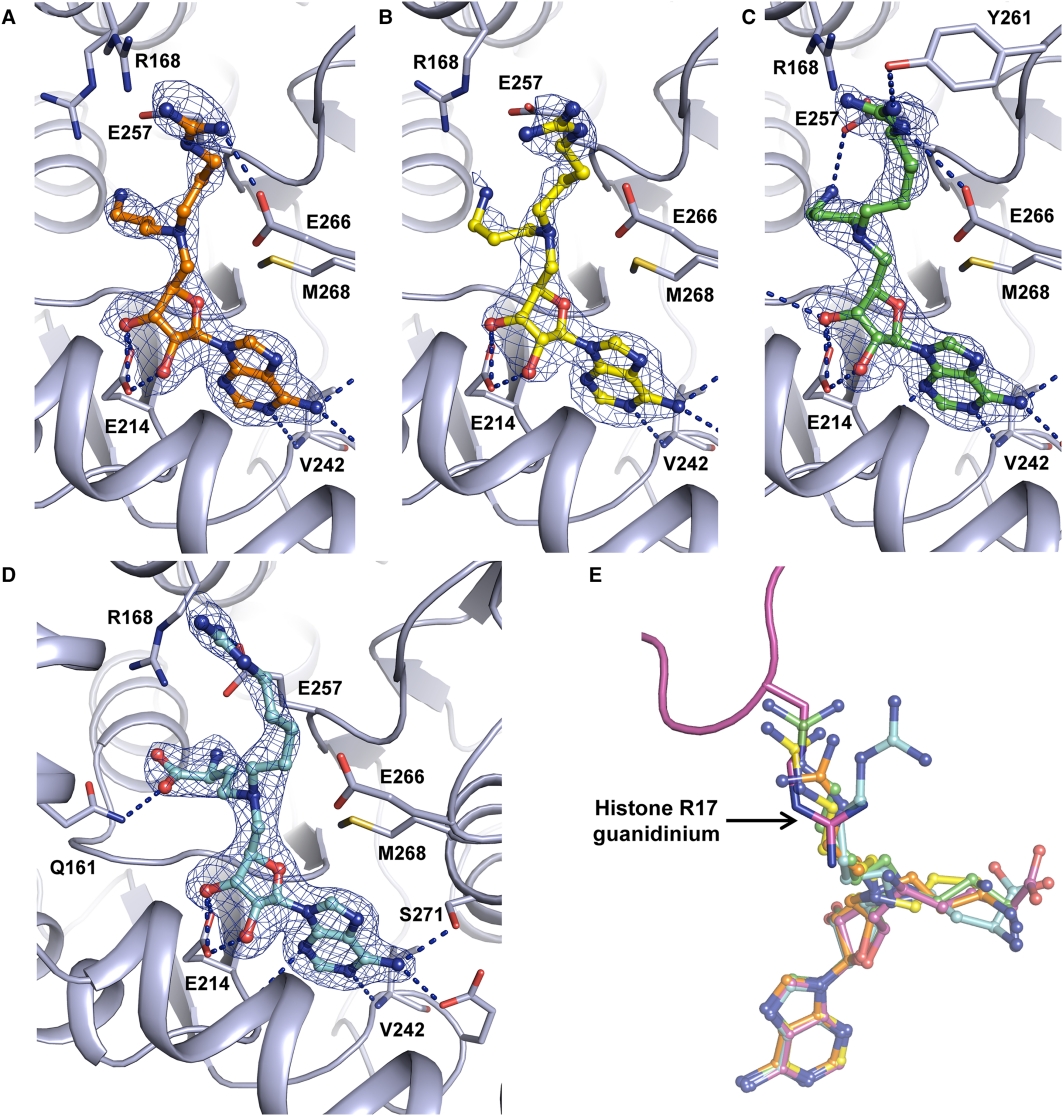
Crystal structures of inhibitors 4, 5, 6 and 7 in complex with CARM1. (**A–D**) 2mF_o_-DF_c_ electron density maps contoured at σ = 1 for **4** (**a**), **5** (**b**), **6** (**c**) and **7** (**d**) in ball-and-stick representation bound in the CARM1 active site. Polar contacts assigned using PyMol are indicated with blue dashed lines and residues involved in SAM and substrate arginine binding (and those involved in putative polar contacts) are shown. (**E**) Superposition of **4** (orange), **5** (yellow), **6** (green) and **7** (cyan) structures as bound to CARM1 with a published structure of CARM1 in complex with histone 3 and sinefungin (magenta, PDB code 5DX0 [48]).

Electron density corresponding to each of these respective inhibitors was clearly observed in the active site (Figure 2A–D). Superposition of the CARM1 complex structures with a published structure of CARM1 in complex with SAM-mimic sinefungin (**3**) and a histone 3 tail substrate (PDB code 5DX0 [48]) revealed that the positions of the adenosine moieties of inhibitors **4**–**7** in the CARM1 active site closely match that of sinefungin (Figure 2E). The alkylguanidinium group also extends into the arginine binding channel as designed, confirming the bisubstrate nature of inhibition. In comparison with the position of H3 Arg-17 (the arginine targeted for CARM1 methylation), the guanidinium groups of all inhibitors reach beyond Glu-257 (which forms a bidentate interaction with the substrate arginine η nitrogens) and Glu-266 (that forms a hydrogen bond to the ε nitrogen of substrate arginines) [48]. The B factors of the guanidinium groups of **4**, **5** and **6** and **7** are relatively high compared with the other ligand atoms, which may suggest that this group retains some flexibility when bound in the active site (Supplementary Figure S3).

### Substitution of the guanidinium group for a 2-aminopyridine improves binding to CARM1 but not PRMT1

Inhibitors **8**, **9** and **10** (Figure 1B) were synthesised with a view to replacing the guanidinium group with less polar 2-aminopyrimidine (**8**) or 2-aminopyridine (**9**, **10**) isosteres. Binding affinities for these molecules with CARM1 (*K*_d_ of 2.30 µM (**8**), 1.14 µM (**9**) and 1.11 µM (**10**), Table 1; Supplementary Figure S4; Supplementary Table S3) were found to be comparable with that obtained for guanidine inhibitor **4** (*K*_d_ 3.32 µM). An activity based assay returned IC_50_ values about five-fold lower (0.7 µM (**8**), 0.3 µM (**9**), 0.8 µM (**10**)), than the guanidinium substituted analogue (2.3 µM for **4**), Table 1 and Supplementary Figure S5. The same molecules (**8**–**10**) were ∼30-fold less potent inhibitors of PRMT1.

**Figure 3.**
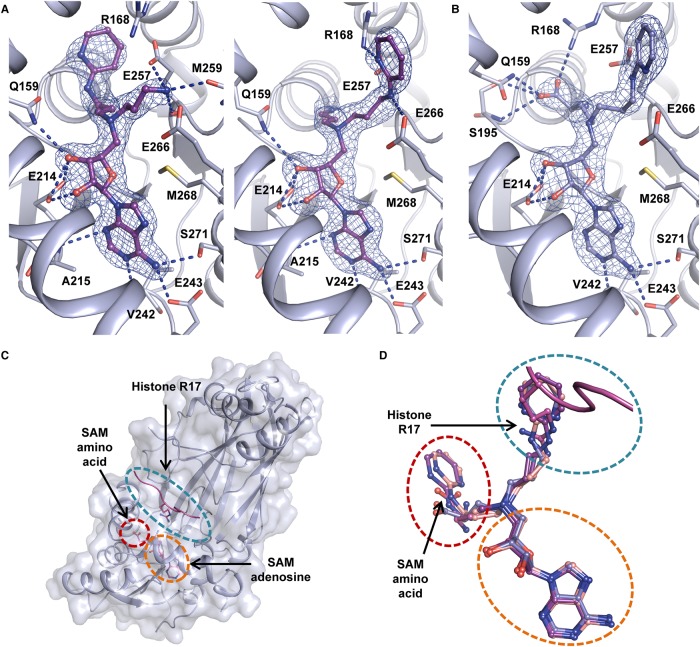
Crystal structures of inhibitors 9 and 10 in complex with CARM1. (**A**) 2mF_o_-DF_c_ electron density maps contoured at σ = 1 for two monomers within the asymmetric unit of the CARM1·**9** complex structure. Polar contacts as computed by PyMol are indicated by blue dashed lines and residues involved in SAM and substrate arginine binding are shown in stick representation and are labelled; (**B**) 2mF_o_-DF_c_ electron density map of inhibitor **10** contoured at σ = 1. Polar contacts and relevant residues are shown as for (**A**). All monomers within the asymmetric unit of the CARM1·**10** structure have similar electron density for **10** corresponding to the same ligand conformation as depicted in (**B**). (**C**) binding pockets of adenosine (orange dashes) and SAM amino acid (red dashes), and substrate binding channel (teal dashes) generated using a CARM1·H3 tail peptide·sinefungin complex crystal structure (PDB code 5DX0) [48]. (**D**) Superposition of complex structures with ligands **8** (pale pink, two conformations observed are shown), **9** (purple, two conformations observed are shown) and **10** (blue, single conformation) with a structure of CARM1 in complex with histone 3 and sinefungin (magenta, PDB code 5DX0) [48].

To gain insight into the binding mode of these ligands, **8**, **9** and **10** were co-crystallised with CARM1 and the resulting structures solved by molecular replacement. Examination of active site electron density maps showed that in all cases the position of the adenosine moiety was the same as observed earlier in structures of CARM1 in complex with **4**–**7** (Figure 3A,B). It was apparent that inhibitors **8** and **9** occupy two conformations within the CARM1 active site, in which the amine and aromatic groups alternately occupy the binding channels of the substrate arginine and the SAM amino acid (Figure 3A,D). Interestingly, introduction of a carboxylate group (as in **10**) resulted in a clear preference for one binding pose within the active site (Figure 3B). This may be attributed to an interaction between Arg-168 and the inhibitor carboxylate, which mimics the carboxylate group of SAM. ITC experiments with **10** revealed that introduction of this carboxylate group does not affect the affinity for CARM1 (Table 1; Supplementary Figure S4; Supplementary Table S3).

No binding of **10** to PRMT1 directly (IC_50_ of 25.3 µM) was observed using ITC under the same conditions as for the other inhibitors (Table 1). To exclude the possibility that **10** inhibits PRMT1 through nonspecific destabilisation, SAH (**2**) was titrated into a solution of PRMT1 and **10**. The presence of **10** in the cell resulted in an increase in the apparent *K*_d_ of SAH (**2**) for PRMT1, while the stoichiometry remained unchanged (Supplementary Table S4). Fitting the resulting isotherm using a competitive binding model thus provided a *K*_d_ ∼ 43.7 µM estimate of **10** for PRMT1 (Supplementary Figure S6).

### CARM1 Asn-265 may contribute to the preferential binding of heteroaromatic inhibitors

**Figure 4.**
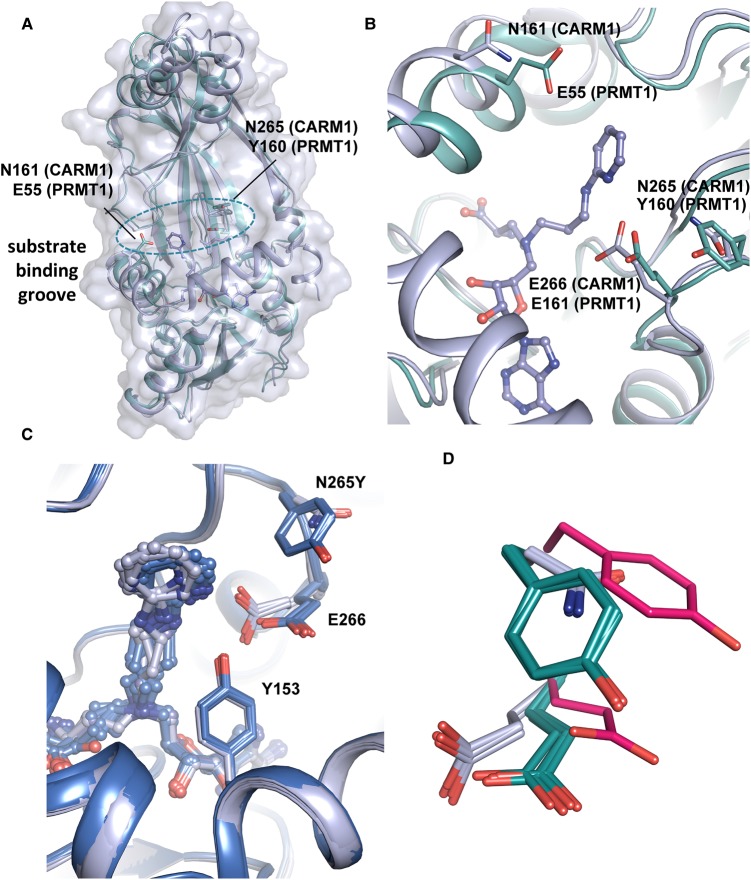
Structural rationale for mutagenesis and crystal structure of 10 with CARM1-N265Y. (**A**) Superposition of CARM1·**10** complex structure (white) with a PRMT1 structure (cyan) in complex with SAH (not shown) (PDB code 1OR8 [50]). The side chains of residues targeted for mutagenesis are shown as sticks. (**B**) Zoomed-in view of position of amino acid sequence differences between PRMT1 and CARM1 investigated. (**C**) Superposition of CARM1·**10** complex structures with (blue) and without (white) the N265Y mutation. Every monomer within the asymmetric unit has been superposed. (**D**) Superposition of all CARM1(WT) monomer structures (white), all CARM1-N265Y monomers (teal) and PRMT1 (PDB code 1OR8, pink [50]).

To explore differences between the CARM1 and PRMT1 active sites, attention focused on two residues in the CARM1 substrate-arginine binding channel (Figure 4). The first, Asn-161 (Glu-55 in PRMT1), was identified as a possible cause for preferential binding of 5C amino acid/guanidine inhibitor **7** to PRMT1 observed previously [34]. A difference in sequence at position CARM1 Asn-265, which corresponds to Tyr-160 in PRMT1 was identified by analysing structural alignments of PRMT1 and CARM1. We hypothesised that the residue in this position may affect the conformation of neighbouring glutamate Glu-266 (Glu-161 PRMT1), which is critical for orienting the substrate arginine residue for methylation and is in close proximity to the aromatic rings in compounds **9** and **10**. This glutamic acid residue adopts a different conformation in CARM1 compared with PRMT1 crystal structures (Figure 4B,D). CARM1 Asn-265 may therefore affect the Glu-266 conformation and, as a consequence, its interactions with the aromatic rings in compounds **9** and **10**. In order to evaluate the possible effects of these two sequence differences CARM1 residues Asn-161 and Asn-265 were mutated into the corresponding PRMT1 residues, Glu and Tyr located at equivalent positions, respectively (Figure 4).

The mutation CARM1 N161E renders the rim of the substrate arginine binding pocket more like PRMT1. Inhibitors (**8**, **9**, **10** and SAH) showed marginally greater affinity (*K*_d_ 0.133–1.11 µM) for the CARM1 N161E mutant compared with wild-type (WT) (0.774–2.30 µM, Table 2; Supplementary Figures S7, S8). The second mutation, CARM1 N265Y renders the substrate arginine-binding pocket more PRMT1-like. A modest decrease in the affinity of **8**, **9** and SAH for CARM1 N265Y compared with WT was observed while the *K*_d_ increased substantially from 1.11 to 143 µM for inhibitor **10** (Table 2; Supplementary Figure S8). These observations reinforce the notion that interactions between the aminopyridine and substrate binding channel are responsible for the preferential binding of **10** to CARM1 over PRMT1. To investigate the structural origin of this effect, CARM1 N265Y was co-crystallised with **10**. As hypothesised the resulting structure revealed a conformational change in Glu-266, in which the glutamate adopts a conformation whereby the plane of the carboxylate group is almost parallel to the phenol side chain of Tyr-265 (Figure 4C,D).

**Table 2:** *K*_d_ values for isosteric inhibitors and 5C amino acid 7 against CARM1 single site PRMT1-mimicking mutants N161E and N265Y measured using ITC

CARM1	*K*_d_ ± standard error from curve fitting (µM)				
	**7**	**8**	**9**	**10**	**SAH**
WT	38.1 ± 9.61	2.30 ± 0.162	1.14 ± 0.411	1.11 ± 0.132	0.774 ± 0.111
N161E	8.18 ± 0.482	1.11 ± 0.201	0.354 ± 0.034	0.181 ± 0.017	0.133 ± 0.022
N265Y	-^1^	5.18 ± 1.86	3.26 ± 0.458	143 ± 75.9^2^	1.49 ± 0.063

1 Not tested.

2 Stoichiometry fixed to 1. Low sigmoidicity makes this value unreliable.

## Discussion

In this study, the binding of a novel series of non-peptidic bisubstrate inhibitors against both PRMT1 and CARM1 has been characterised. Unexpectedly, the length of a methylene linker between the guanidinium and azo adenosylmethionine moieties that comprise a subset of these inhibitors was not a determining factor for PRMT1 and CARM1 affinity, nor the position of the guanidinium group within the active site of CARM1. On testing an analogous series of amino acid-guanidine inhibitors, we previously observed a significant reduction in affinity for CARM1 on increasing the alkyl linker length from 3 to 5 methylene units [34]. In contrast with previous results, a weak interaction of the 5C amino acid-guanidine **7** with both PRMT1 and CARM1 was detected, exhibiting IC_50_ and *K*_d_ values of 20–40 µM. Binding of **7** to CARM1 is also evidenced by the clear electron density in a co-crystal structure (Figure 2D). The apparent difference between these results and those previously reported regarding inhibitor **7** may be a result of the different CARM1 constructs used. In order to be able to compare trends from the measured dissociation constants by ITC and IC_50_ values we focused on the catalytic core of CARM1 [34]. It has been reported that indole and pyrazole CARM1 inhibitors displayed different relative potency when tested using an activity assay with GST-tagged full-length protein and ITC with the catalytic core [41]. It is possible that the presence of the GST-tag or additional regions of CARM1 outside of the catalytic core influence inhibitor binding. Elucidating a structure of full-length CARM1 in complex with inhibitors or its substrates, and determining the precise role of the domains flanking the catalytic core, will aid the design of CARM1 inhibitors. The bisubstrate nature of the inhibitors described herein necessitates care in interpretation of IC_50_ data. In the absence of detailed kinetic analysis (allowing conversion of IC_50_ values to *K*_i_s) an interpretation of selectivity of an inhibitor between different enzymes based on IC_50_ data [49] has to be treated with caution. Since such analysis is beyond the scope of the present study, *K*_d_ measurements (which do not suffer this complication) were obtained to enable direct comparison of the affinity of the inhibitor series for PRMT1 *versus* CARM1.

While these experiments were being conducted, binding studies on a related inhibitor series were reported. [28,33] Comparing the trends of reported IC_50_ values suggests that the optimal number of atoms between the 4′ ribose carbon and the guanidine group is three (equivalent to a 1 methylene linker in our inhibitors), or two atoms joined by a double bond. The differing assay conditions and inhibitor structures mean that these values cannot be directly compared with ours. Nonetheless, the high potency achieved with these shorter linker lengths, in combination with the inhibition and crystallographic data reported herein (Figure 2), suggest that the 3 to 5 methylene alkylguanidines in our inhibitors could be shortened to a methylguanidine group to better align the inhibitor guanidine for interaction with CARM1's active site glutamates, thus improving binding.

Both, inhibitors **9** and **10** displayed preferential binding for CARM1 over PRMT1. For **10**, a *K*_d_ estimate of 43.7 µM for PRMT1 (IC_50_ of 25.3 µM) was determined through a competition binding experiment as direct binding upon titrating **10** into PRMT1 was not observed, presumably because the heat change associated with ligand binding was below the limit of detection). This *K*_d_ value is broadly consistent with the IC_50_ value, and indicates that the inhibitors **9** and **10** binding more strongly to CARM1 over PRMT1 by ∼15- and ∼40-fold, respectively (as judged by ITC). This degree of selectivity provides a promising framework for the development of higher potency chemical probes for cellular assays. Interestingly, nine structures have recently been added to the PDB of CARM1 from *Mus musculus* in complex with different aromatic-containing bisubstrate inhibitors (PDB codes 5TBJ, 5TBI, 5TBH, 5LV3, 5LV2 [36] and 5ISB 5IS9, 6DVR and 6D2L [38]). Superposition of these structures with the CARM1–**9** complex structure (Supplementary Figure S9) revealed reasonably good overlap of **9**’s aminopyridine group with the aromatic groups of these inhibitors, particularly in the SAM carboxylate binding pocket (i.e. superposition with SKI-72, Supplementary Figure S9). The presence of aromatic groups in this pocket further supports our finding that **8** and **9** adopt alternate conformation in which the aromatic group may occupy either the substrate binding channel or the SAM carboxylate binding pocket (Figure 3). The observed trend towards increased potency for CARM1 by using hydrophobic guanidine isosteres will be useful in the pursuit of additional CARM1 chemical probes.

**Figure 5.**
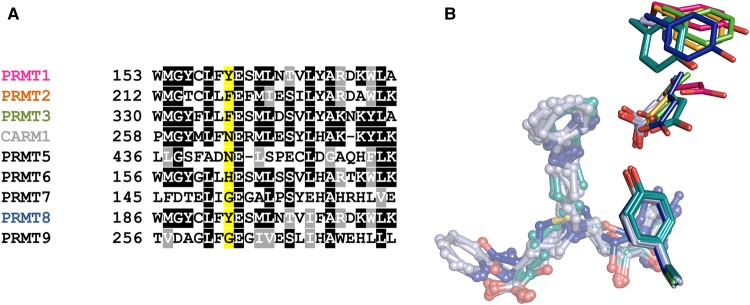
Sequence conservation of CARM1-N265 across other PRMTs and position of substrate arginine-interacting glutamic acid residue in PRMT structures. (**A**) Sequence alignment of human PRMT active sites showing site of N265Y mutation generated using ClustalW and Box Shade. The site targeted for mutagenesis is highlighted in yellow. Sites that show >50% consensus are shaded (black for identical residues and grey for similar residues). Colour coding matches the structures shown in (**B**). (**B**) Superposition of PRMT1 (1OR8, hot pink [50]), PRMT2 (5JMQ, yellow), PRMT3 (1F3L, green [51]) and PRMT8 (5DST, blue [52]) with CARM1 (WT) (bound to **9**, light grey, all monomers in the asymmetric unit shown) and CARM1-N265Y (bound to **10**, teal), which all have a tyrosine or phenylalanine adjacent to the active site glutamate.

Mutagenesis studies revealed that CARM1 Asn-265 may be important for the binding of inhibitors with hydrophobic guanidine isosteres (**9** and **10**). The effect of this mutation on the position of Glu-266 in the crystal structure of CARM1 N265Y is notable. It has been suggested that the corresponding glutamate (Glu-161) in PRMT1 [50] is catalytically incompetent since it appears to be rotated away from the active site (PDB code 1OR8) [48,50]. This was attributed to likely protonation of Glu-161 due to the low pH at which the crystals formed. However, these studies reveal that substitution of CARM1 Asn-265 with a tyrosine, as present in PRMT1, results in a conformation of the glutamate side chain similar to PRMT1 in crystals grown at a pH of 7.0. This suggests that this alteration in the glutamate conformation is predominantly a result of interaction with the neighbouring tyrosine, rather than protonation of the side chain carboxylate. A sequence alignment of human PRMTs (Figure 5A) reveals that PRMTs 1, 2, 3, 6 and 8 have aromatic side chains in this position (Y, F, F, H, Y, respectively), while PRMTs 4, 5, 7 and 9 have non-aromatic side chains (N, N, G, G, respectively). A superposition of available PRMT structures with tyrosine or phenylalanine adjacent to the conserved glutamate (Figure 5B) shows that PRMT8, which has a tyrosine in this position exhibits a conformation of the neighbouring glutamate similar to that seen in the CARM1-N265Y mutant. The glutamate side chains of PRMT2 and PRMT3 (which have a phenylalanine at this position) are closer to that seen in WT CARM1. Further investigation will be required to determine the extent to which these sequence differences can be exploited for selective PRMT inhibitor development.

Crystal structures (Figure 3) show that inhibitors **8** and **9**, which feature terminal primary amines, may adopt an alternative binding conformation that positions the aminopyridine group into the subsite that normally binds the amino acid terminus of SAM. In contrast, inhibitor **10** has an amino acid terminus and adopts a single conformation that places the aminopyridine in the arginine-binding channel. This suggests that the inhibitor carboxylate group may be required to ensure the arginine mimic occupies the desired pocket. Despite conferring this apparent conformational preference, the carboxylate group does not appear to enhance the affinity of our series of inhibitors for CARM1, with **9** and **10** displaying similar *K*_d_ and IC_50_ values (Table 1). Furthermore, addition of a carboxylate group to 5C guanidine-amine **3** (i.e. inhibitor **4**) resulted in a slight decrease in potency. A terminal amino acid group (as in **7** and **10**), or appropriate isosteric replacement may therefore be beneficial for ensuring that the arginine mimicking group exclusively occupies the substrate binding channel in a single binding mode, as achieved by SAM for catalysis.

Overall, these results demonstrate effective incorporation of isosteric replacement of guanidine for a less polar 2-aminopyridine into bisubstrate PRMT inhibitors. Investigation of further isosteric replacements will be required to achieve greater rigidity, potency and cell permeability displayed by current lead compounds. Different binding for these inhibitors between CARM1 and PRMT1 prompted structural investigation which revealed differences at equivalent positions in the substrate channels for these respective enzymes (i.e. Asn-265 in CARM1 or Tyr-160 in PRMT1). Further investigation of binding and potency for bisubstrate inhibitors with other PRMTs that feature these differences will help determine whether they can be exploited to confer selectivity to future inhibitor designs.

## Data Availability

Atomic co-ordinates and structure factors were deposited in the Protein Data Bank (http://wwpdb.org/) under accession IDs 6S7C, 6S7B, 6S70, 6S71, 6S74, 6S7A, 6S79 and 6S77.

## Abbreviations

aDMA: asymmetric dimethylarginine
AML: acute myeloid leukemia
Bis-tris propane: 1,3-Bis[tris(hydroxymethyl)methylamino]propane
BSA: bovine serum albumin
CARM1: co-activator associated arginine methyltransferase 1
DPM: disintegrations per minute
GST: glutathione-S-transferase;
IC_50_: inhibitor concentration giving half maximal inhibition
ITC: isothermal titration calorimetry
*K*_d_: dissociation constant
*K*_i_: inhibition constant
MBP: maltose binding protein
MMA: monomethyl arginine
PDB: Protein data bank
PRMT: protein arginine methyltransferase
SAH: *S*-Adenosylhomocysteine
SAM: *S*-Adenosylmethionine
sDMA: symmetric dimethylarginine
TEV: tobacco etch virus
WT: wild-type
