# Supplementary material for: Structural and biochemical evaluation of bisubstrate inhibitors of protein arginine N-methyltransferases PRMT1 and CARM1 (PRMT4)
Source: Biochem J. 2020 Feb 27;477(4):787–800. doi: 10.1042/BCJ20190826 (PMC7054760; doi:10.1042/BCJ20190826)
Supplement: Supplementary Tables S1-S4 and Figures S1-S9 [file BCJ-477-787-s1.pdf]

Supplementary Material

Supplementary experimental data for compounds 5, 6, 8, 9 and 10

5 - 1-(4-((((2*R*,3*S*,4*R*,5*R*)-5-(6-amino-9*H*-purin-9-yl)-3,4-dihydroxytetrahydro furan-2-yl)methyl)(3-aminopropyl)amino)butyl)guanidine hydrochloride: $[\alpha]^{23}_D$ 17.66; ν_{max}/cm^{-1} 3317, 3167, 1683, 1051; ^{1}H NMR (400 MHz, D_2O) δ_H 8.46 (s, 1H, Ad-*H*), 8.44 (s, 1H, Ad-*H*), 6.14 (d, *J*=4.0 Hz, 1H, 1'-*H*), 4.79 (m, 1H, 2'-*H*), 4.44-4.52 (m, 2H, 3'-*H* and 4'-*H*), 3.73-3.79 (1H, m, 5'-CH_aH$_b$), 3.67 (dd, *J*=14.0, 1.6, 1H, 5'-CH$_a$$H_b$), 3.35 (t, *J*=8.1, 2H, CH_2), 3.29 (t, *J*=8.3, 2H, CH_2), 3.02-3.06 (m, 4H, CH_2 and CH_2), 2.08-2.15 (m, 2H, CH_2), 1.71-1.73 (m, 2H, CH_2), 1.52-1.54 (m, 2H, CH_2); ^{13}C NMR (100 MHz, D_2O) δ_C 156.6, 150.1, 148.1, 144.7, 143.4, 119.1, 89.9, 78.0, 73.1, 71.5, 54.7, 53.0, 50.5, 40.4, 36.6, 24.9, 21.5, 20.2. HRMS (ESI+) m/z: (M+H $^+$) calcd $C_{18}H_{33}N_{10}O_3^+$ 437.2737, found 437.2731.

6 - 3-((4-((amino(iminio)methyl)amino)butyl)(((2*R*,3*S*,4*R*,5*R*)-5-(6-amino-9*H*-purin-9-yl)-3,4-dihydroxytetrahydrofuran-2-yl)methyl)amino)propan-1-aminium chloride $[\alpha]^{23}_D$ +23.55 (c 1.0 in H_2O); ν_{max}/cm^{-1} 3363, 1647, 1436, 1120. ^{1}H NMR (400 MHz, D_2O) δ_H: 8.42 (s, 1H, Ad-*H*), 8.40 (s, 1H, Ad-*H*), 6.12 (d, *J*=4.0 Hz, 1H, 1'-*H*), 4.80-4.51 (m, 1H, 2'-*H*), 4.43-4.51 (m, 2H, 3'-*H* and 4'-*H*), 3.72-3.78 (1H, m, 5'-CH_aH$_b$), 3.66 (d, *J*=14.0 Hz, 1H, 5'-CH$_a$$H_b$), 3.32 (t, *J*=7.8, 2H, CH_2), 3.24 (t, *J*=7.9, 2H, CH_2), 3.00-3.04 (m, 4H, CH_2 and CH_2), 2.08-2.15 (m, 2H, CH_2), 1.65-1.75 (m, 2H, CH_2), 1.40-1.45 (m, 2H, CH_2), 1.24-1.28 (m, 2H, CH_2); ^{13}C NMR (100 MHz, D_2O) δ_C: 156.6, 150.7, 148.2, 145.7, 143.1 , 119.2, 89.9, 78.0, 73.0, 71.6, 54.8, 53.3, 50.5, 40.8, 36.5, 27.3, 23.6, 22.8, 21.5. HRMS (ESI+) m/z: (M+H$^+$) calcd $C_{19}H_{35}N_{10}O_3^+$ 451.2894, found 451.2901.

8 - 3-((((2R,3S,4R,5R)-5-(6-amino-9H-purin-9-yl)-3,4-dihydroxytetrahydrofuran-2-yl)methyl)(3-(pyrimidin-2-ylamino)propyl)amino)propan-1-aminium chloride: $[\alpha]^{23}_D$ +23.55 (c 1.0 in H_2O); ν_{max}/cm^{-1} 3084, 1644, 1052; ^{1}H NMR (400 MHz, D_2O) δ_H: 8.09 (s, 1H, Ad-H), 8.01 (s, 1H, Ad-H), 7.87 (d, *J*= 4.9, 2H, Ar-H), 6.31 (app. t, *J*= 4.9, 1H, Ar-H), 5.85 (d, *J*= 4.8, 1H, 1'-H), 4.66 (app. t, *J* = 4.8, 1H, 2'-H), 4.22-4.23 (m, 1H, 3'-H), 4.15 (app. t, *J*= 5.3, 1H, 4'-H), 3.01-3.06 (m, 1H, CH_aH$_b$), 2.93-2.98 (m, 1H, CH$_a$$H_b$), 2.95 (t, *J*= 7.3, 2H, CH_2), 2.81-2.87 (1H, m, 1H, 5'-CH_aH$_b$), 2.73 (d, *J*=14.4, 1H, 5'-CH$_a$H$_b$), 2.61-2.67 (m, 2H, CH_2), 2.50-2.56 (m, 2H, CH_2), 1.72-1.79 (m, 2H, CH_2), 1.61-1.66 (m, 1H, CH_aH$_b$), 1.52-1.58 (m, 1H, CH$_a$$H_b$); ^{13}C NMR (100 MHz, D_2O) δ_C: 160.4, 157.7, 155.1, 152.5, 148.4, 139.8, 118.6, 109.9, 87.9, 81.4, 72.9, 72.0, 55.4, 51.7, 51.5, 39.4, 38.5, 24.4, 23.8; HRMS (ESI+) m/z: (M+H$^+$) calcd $C_{20}H_{31}N_{10}O_3^+$ 459.2581, found 459.2575.

9 - 3-((((2*R*,3*S*,4*R*,5*R*)-5-(6-amino-9*H*-purin-9-yl)-3,4-dihydroxytetrahydrofuran-2-yl)methyl)(3-(pyridin-2-ylamino)propyl)amino)propan-1-aminium chloride: $[\alpha]^{23}_D$ +7.40 (c 1.0 in H_2O); ν_{max}/cm^{-1} 3475, 3009, 1465, 1016; ^{1}H NMR (400 MHz, D_2O) δ_H: 8.40 (s, 1H, Ar-*H*), 7.71-7.76 (m, 1H, Ar-*H*), 7.61 (d, *J*=6.5, 1H, Ar-H), 6.83-6.86 (m, 1H, Ar-*H*), 6.75 (app t, *J*=6.5, 1H, Ar-H), 6.05 (d, *J*=3.6, 1H, 1'-*H*), 4.61 (app t, *J*= 3.6, 1H, 2'-*H*), 4.43-4.44 (m, 2H, CH and C*H*aHb), 3.80-3.82 (m, 1H, CH), 3.68 (d *J*=14.3, 1H, CH$_a$$H_b$), 3.63 (1H, td, *J*=6.1, 2.6, CH) 3.35-3.42 (m, 4H, and 2xCH_2), 3.00-3.12 (m, 3H, CH_2 and CH_aCH$_b$), 2.00-2.13 (m, 4H, 2xCH_2); ^{13}C NMR (100 MHz, D_2O) δ_C: 152.3, 149.8, 147.8, 144.5, 143.8, 143.2, 134.8, 119.1, 112.7, 90.3, 77.5, 73.2, 71.4, 58.9, 51.10, 51.07, 48.9, 38.6, 36.4, 21.8, 21.3; HRMS (ESI+) m/z: (M+H$^+$) calcd $C_{21}H_{31}N_9O_3^+$ 458.2623, found 458.2623.

10 (*S*)-4-((((2*R*,3*S*,4*R*,5*R*)-5-(6-amino-9*H*-purin-9-yl)-3,4-dihydroxytetrahydrofuran-2-yl)methyl)(3-(pyridin-2-ylamino)propyl)amino)-2-ammoniobutanoate, chloride salt**:** $[\alpha]^{23}_D$ +20.73 (c 1.0 in H_2O); ν_{max}/cm^{-1} 3441 (N-H), 3007 , 1623, 1240, 1015; ^{1}H NMR (400 MHz, D_2O) δ_H 8.43 (s, 1*H*, adenosine-*H*), 8.39 (s, 1H, adenosine-*H*), 7.75-7.79 (m, 1H, Pyr*H*), 7.65 (d, *J*=6.2, 1H, Pyr*H*), 6.88 (d, *J*= 8.5, 1H, Pyr*H*), 6.76-6.80 (m, 1H, Pyr*H*), 6.09 (d, *J*=3.6, 1H, 1'-*H*), 4.64-4.66 (m, 1H, 2'-*H*), 4.42-4.45 (m, 2H, C*H,* CH$_a$$H_b$), 4.08-4.14 (m, 1H, C*H*), 3.82-3.90 (m, 1H, C*H*), 3.72 (d, J=13.6, 1H, CH$_a$$H_b$), 3.29-3.63 (m, 6H, C$H_2$), 2.38-2.48 (m, 1H, C$H_aCH_b$CH), 2.26-2.33 (m, 1H, CH$_a$CH_b), 2.02-2.09 (m, 2H, CH_2C$H_2$$CH_2$), ^{13}C NMR (100 MHz, D_2O) δ_C 170.4, 152.2, 149.7, 147.8, 144.4, 143.8, 143.2, 134.8, 119.1, 112.7, 90.3, 78.0, 73.1, 71.4, 54.7, 51.1, 50.9, 50.8, 50.5, 38.6, 24.1, 21.9; HRMS (ESI+) *m/z*: (M+H$^+$) calcd $C_{22}H_{31}N_9O_5^+$ 502.2520, found 502.2521.

Supplementary Tables

Inhibitor	[CARM1]/mg/mL	Crystallisation condition
**4**	1.7	0.1 M Bis-tris propane pH 7.0, 0.02 M sodium phosphate, 21 % (w/v) PEG 3350
**5**	1.7	0.1 M Bis-tris propane pH 7.0, 0.02 M potassium phosphate, 22 % (w/v) PEG 3350
**6**	1.7	0.1 M Bis-tris propane pH 7.0, 0.2 M sodium acetate, 24 % (w/v) PEG 3350
**7**	1.2	0.1 M Bis-tris propane pH 7.0, 0.2 M sodium acetate, 20 % (w/v) PEG 3350
**8**	1.0	0.1 M Bis-tris propane pH 7.0, 0.02 M sodium phosphate, 22 % (w/v) PEG 3350
**9**	3.0	0.1 M Bis-tris propane pH 8.5, 0.2 M sodium formate, 20 % (w/v) PEG 3350
**10**	3.0	0.1 M Bis-tris propane pH 8.5, 0.2 M sodium formate, 20 % (w/v) PEG 3350
**10** w/CARM1 N265Y	2.4	0.1 M Bis-tris propane pH 7.0, 0.02 M sodium phosphate, 26 % (w/v) PEG 3350

Table S1. Crystallisation conditions for CARM1-inhibitor complexes.
The protein concentrations indicated are the concentrations measured prior to addition of a half-volume of 500 µM ligand solution, then mixing with the well solution, as detailed in the main text.

	4	5	6	7	8	9	10	10 (CARM1 N265Y)
**PDB Code**	6S7C	6S7B	6S70	6S71	6S74	6S7A	6S79	6S77
**Beamline**	ID30B (ESRF)[1]	I02 (DLS)[2]	ID30B (ESRF)	ID30B (ESRF)	ID30B (ESRF)	I03 (DLS)	I03 (DLS)	ID30-A1 (ESRF)
**Wavelength (Å)**	0.9762	0.9795	0.9762	0.9762	0.9762	0.9762	0.9762	0.9800
**Resolution range**	51.86 - 2.30 (2.38 - 2.30)	61.12 - 2.66 (2.75 - 2.66)	57.23 - 2.30 (2.38 - 2.30)	88.99 - 2.06 (2.14 - 2.06)	48.17 - 2.1 (2.18 - 2.1)	71.42 - 1.86 (1.93 - 1.86)	69.48 - 2.10 (2.18 - 2.10)	48.01 - 2.12 (2.20 - 2.12)
**Space group**	P 21 21 2	P 21 21 2	P 21 21 2	P 21 21 2	P 21 21 2	P 21 21 2	P 21 21 2	P 21 21 2
**Unit cell a (Å)**	74.697	75.455	74.684	74.72	75.393	74.784	75.479	74.988
**b (Å)**	98.629	99.367	98.677	98.63	99.029	98.624	99.196	98.718
**c (Å)**	207.438	208.463	207.1	206.36	207.952	207.137	208.439	207.071
**Total reflections**	279649 (18740)	294369 (2801)	276271 (17387)	342936 (15243)	302969 (16836)	852511 (33682)	612585 (30578)	350341 (18242)
**Unique reflections**	66343 (4477)	45903 (437)	66028 (4361)	91634 (4501)	80206 (4644)	129298 (6329)	92162 (4483)	87715 (4472)
**Multiplicity**	4.2 (4.2)	6.4 (6.4)	4.2 (4.0)	3.7 (3.4)	3.8 (3.6)	6.6 (5.3)	6.6 (6.8)	4.0 (4.1)
**Completeness (%)**	96.7 (98.2)	99.9 (98.0)	96.2 (95.8)	96.7 (96.5)	87.8 (90.1)	100.0 (100.0)	100.0 (99.9)	99.7 (99.8)
**Mean I/sigma(I)**	11.8 (2.2)	10.1 (2.8)	13.8 (2.4)	11.8 (2.1)	11.6 (2.2)	9.1 (0.8)	7.4 (0.9)	8.8 (1.2)
**R-merge**	0.075 (0.741)	0.210 (1.052)	0.065 (0.546)	0.058 (0.538)	0.064 (0.496)	0.095 (1.829)	0.186 (2.516)	0.096 (1.176)
**R-pim**	0.041 (0.412)	0.090 (0.450)	0.044 (0.295)	0.033 (0.325)	0.034 (0.269)	0.040 (0.876)	0.078 (1.040)	0.054 (0.655)
**$CC_{1/2}$**	0.998 (0.684)	0.994 (0.781)	0.999 (0.765)	0.999 (0.780)	0.999 (0.818)	0.998 (0.393)	0.996 (0.287)	0.998 (0.431)
**Refinement**								
**No of reflections**	66203 (6645)	45808 (4499)	65966 (6428)	91546 (9039)	80113 (8094)	129154 (12752)	92043 (9068)	87609 (8680)
**R-work**	0.2068 (0.3141)	0.1945 (0.3060)	0.1851 (0.2571)	0.2336 (0.3088)	0.1921 (0.2845)	0.1993 (0.3201)	0.1989 (0.3268)	0.2039 (0.3111)
**R-free**	0.2355 (0.3306)	0.2278 (0.3406)	0.2129 (0.2893)	0.2542 (0.3531)	0.2151 (0.3084)	0.2194 (0.3504)	0.2314 (0.3406)	0.2464 (0.3230)
**<B-factors> (Å²)**								
**Protein**	45.09	41.05	42.47	42.01	35.46	45.96	45.82	42.68
**Inhibitors**	50.40	53.48	49.10	57.64	35.74	48.13	47.71	42.39
**RMS bond lengths (Å)**	0.016	0.016	0.020	0.012	0.018	0.015	0.032	0.013
**RMS bond angles (°)**	1.76	1.72	1.73	1.52	1.51	1.66	2.07	1.62

Table S2. Data collection and refinement statistics for CARM1-inhibitor crystal structures. Statistics for the highest-resolution shell are given in parentheses.

Inhibitor	PRMT1			
	ΔG (kJ/mol)	ΔH ± standard error from curve fitting (kJ/mol)	-TΔS (kJ/mol)	N ±standard error from curve fitting
**SAH**	-35.1	-97.7 ± 1.4	62.6	0.485 ± 0.0037
**4**	-28.5	-67 ± 3.22	38.5	0.519 ± 0.0082
**5**	-30.8	-48.1 ± 1.86	17.2	0.549 ± 0.0092
**6**	-28.6	-41.5 ± 2.79	12.9	0.607 ±0.019
**7**	-25.7	-27.5 ± 1.4	1.84	N/A[1]
**8**	-28.5	-43.2 ± 10.5	14.7	0.322 ± 0.028
**9**	-27.5	-29.4 ± 1.16	39.1	0.716 ± 0.012
**10**	-24.9	-6.71[2]	-18.2	N/A[2]
**Inhibitor**	**CARM1**			
	ΔG (kJ/mol)	ΔH ± standard error from curve fitting (kJ/mol)	-TΔS (kJ/mol)	N ±standard error from curve fitting
**SAH**	-34.9	-147 ± 4.62	113	0.549 ± 0.0086
**4**	-31.3	-103 ± 2.65	71.9	0.481 ± 0.0062
**5**	-29.4	-110 ± 2.98	80.5	0.501 ± 0.0060
**6**	-28.6	-79.2 ± 6.42	50.5	0.417 ± 0.012
**7**	-25.2	-77.7 ± 9.65	52.5	0.648 ± 0.032
**8**	-32.2	-124 ± 1.86	92.2	0.529 ± 0.0042
**9**	-34	-86.2 ± 7.42	52.3	0.529 ± 0.025
**10**	-34	-73.1 ± 1.92	39.1	0.536 ± 0.0079

[1] The stoichiometry was fixed to 0.5 due to a low c-value curve.
[2] Data were determined using a competition experiment, therefore the ΔH error and binding stoichiometry are not calculated.

Table S3. Thermodynamic parameters from ITC data for inhibitors 4, 5, 6, 7, 8, 9 and 10 titrations into PRMT1 and CARM1.

ΔG, ΔH, -TΔS and stoichiometry (N) determined from titrations of SAH and inhibitors **4**, **5**, **6**, **7**, **8**, **9**, and **10** into PRMT1 (top) or CARM1 (bottom). Standard errors are not calculated for ΔG and -TΔS since they are calculated indirectly using the best-fit values of K_d and ΔH.

	Inhibitor	CARM1<br>ΔG (kJ/mol)	ΔH ± standard error (kJ/mol)	-TΔS (kJ/mol)	N± standard error
**N161E**	**SAH**	-39.3	-125 ± 1.84	86.1	0.692 ± 0.0047
	**8**	-34	-113 ± 3.79	79.2	0.745 ± 0.012
	**9**	-36.8	-86.7 ± 1.17	49.9	0.914 ± 0.005
	**10**	-38.5	-94.1 ± 0.971	55.6	1.02 ± 0.004
**N265Y**	**SAH**	-33.3	-99.1 ± 1.05	65.9	0.737 ± 0.004
	**8**	-30.2	-62.8 ± 8.41	32.7	0.698 ± 0.051
	**9**	-31.3	-61.9 ± 3.36	30.6	1.07 ± 0.035
	**10**	-[1]	-	-	-

[1] The low sigmoidicity of the CARM1-N265Y/**10** curve was not sufficient for reliable determination of binding parameters.

Table S4. Thermodynamic parameters from SAH and inhibitors 8, 9, and 10 titrations into CARM1 N161E and N265Y mutants.

ΔG, ΔH, -TΔS values derived from ITC data of SAH and inhibitors **8**, **9**, and **10** into CARM1 N161E and N265Y mutants are shown. Standard errors are not calculated for ΔG and -TΔS since they are calculated indirectly using the best-fit values of K_d and ΔH.

Supplementary Figures

(a)

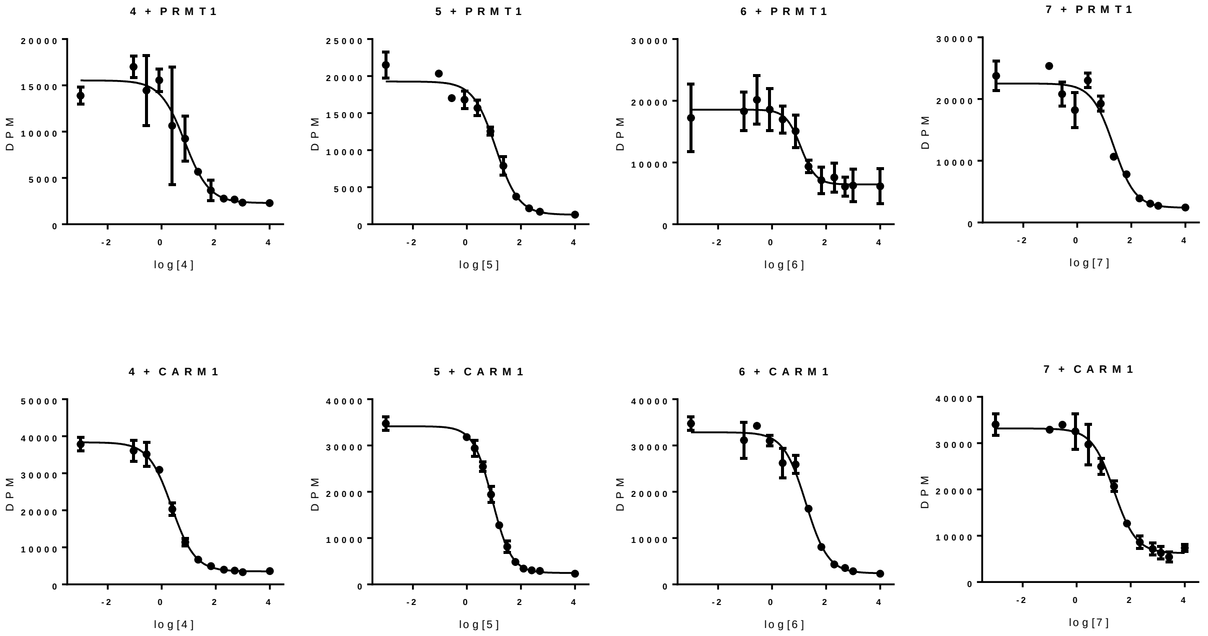

(b)

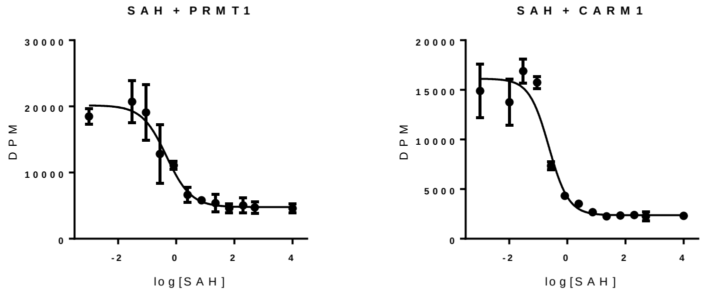

Supplementary Figure S1. Dose-response curves for inhibitors 4, 5, 6, and 7 and SAH.

(a) IC_{50} curves for inhibitors **4**, **5** and **6** and **7** with PRMT1 and CARM1. Error bars indicate standard deviation (S.D.) at each inhibitor concentration (n=2). DPM is the un-normalised disintegrations per minute, measured by liquid scintillation counting, and is proportional to tritium incorporation into the substrate.

(b) IC_{50} curves for SAH with PRMT1 and CARM1. Error bars indicate S.D. at each inhibitor concentration (n=2). DPM is the un-normalised disintegrations per minute, measured by liquid scintillation counting, and is proportional to tritium incorporation into the substrate.

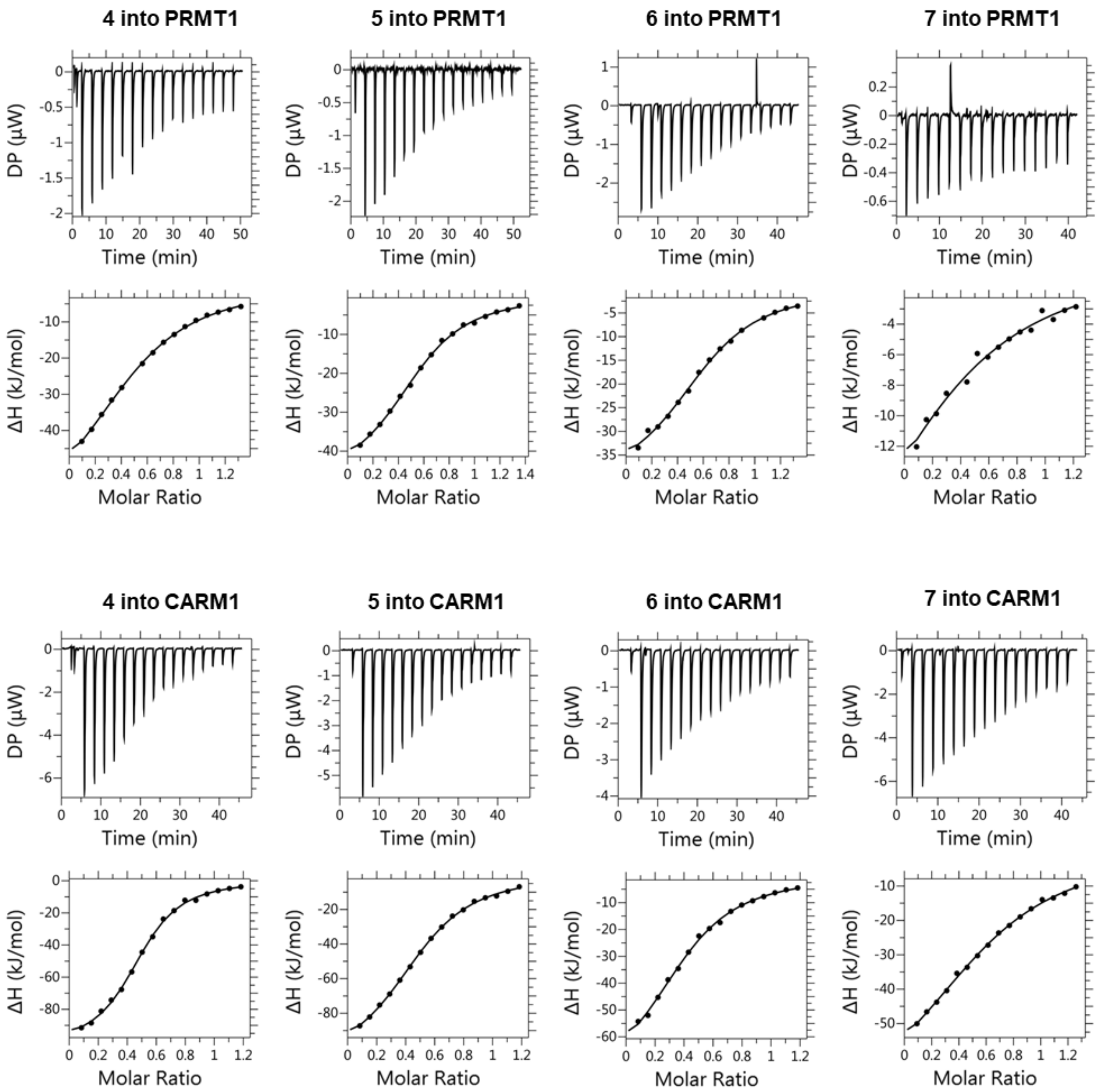

Supplementary Figure S2. ITC thermograms (upper panels) and integrated heat plots (lower panels) for inhibitors 4, 5, 6 and 7.

The inhibitor and PRMT used for each titration are given above the relevant graphs. CARM1 and PRMT1 titrations were conducted at 25°C with PRMT concentrations in the range of 42 – 121 μM and inhibitor concentrations in the range of 230 – 736 μM.

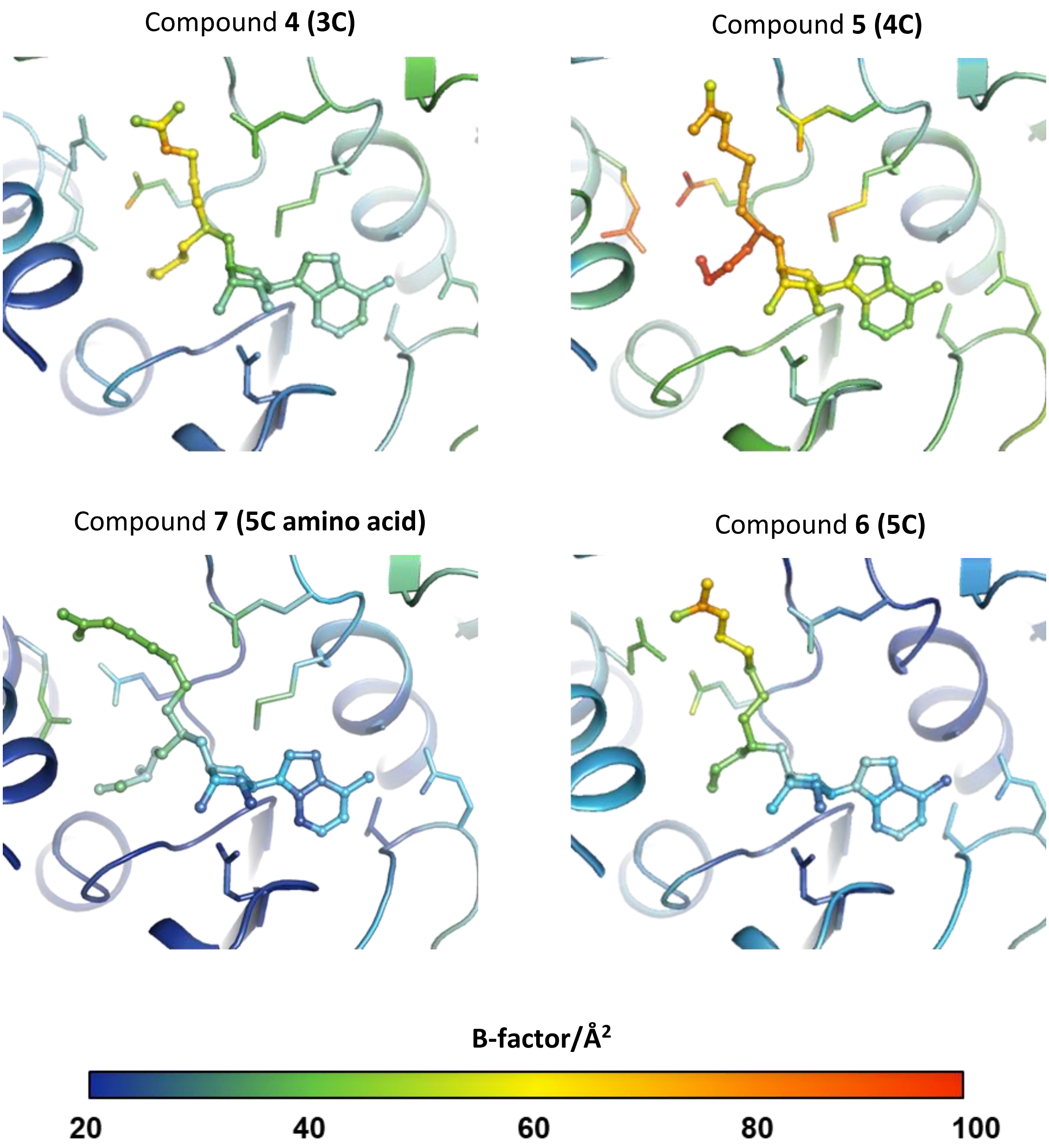

Supplementary Figure S3. Graphical representation of B-factors in respective inhibitor 4, 5, 6 and 7 – CARM1 complex crystal structures.

The structures in cartoon representation with the inhibitors shown in ball-and-stick are coloured according to B-factor values as shown in the spectrum bar below ranging from dark blue (20 Å^2) to red (100 Å^2).

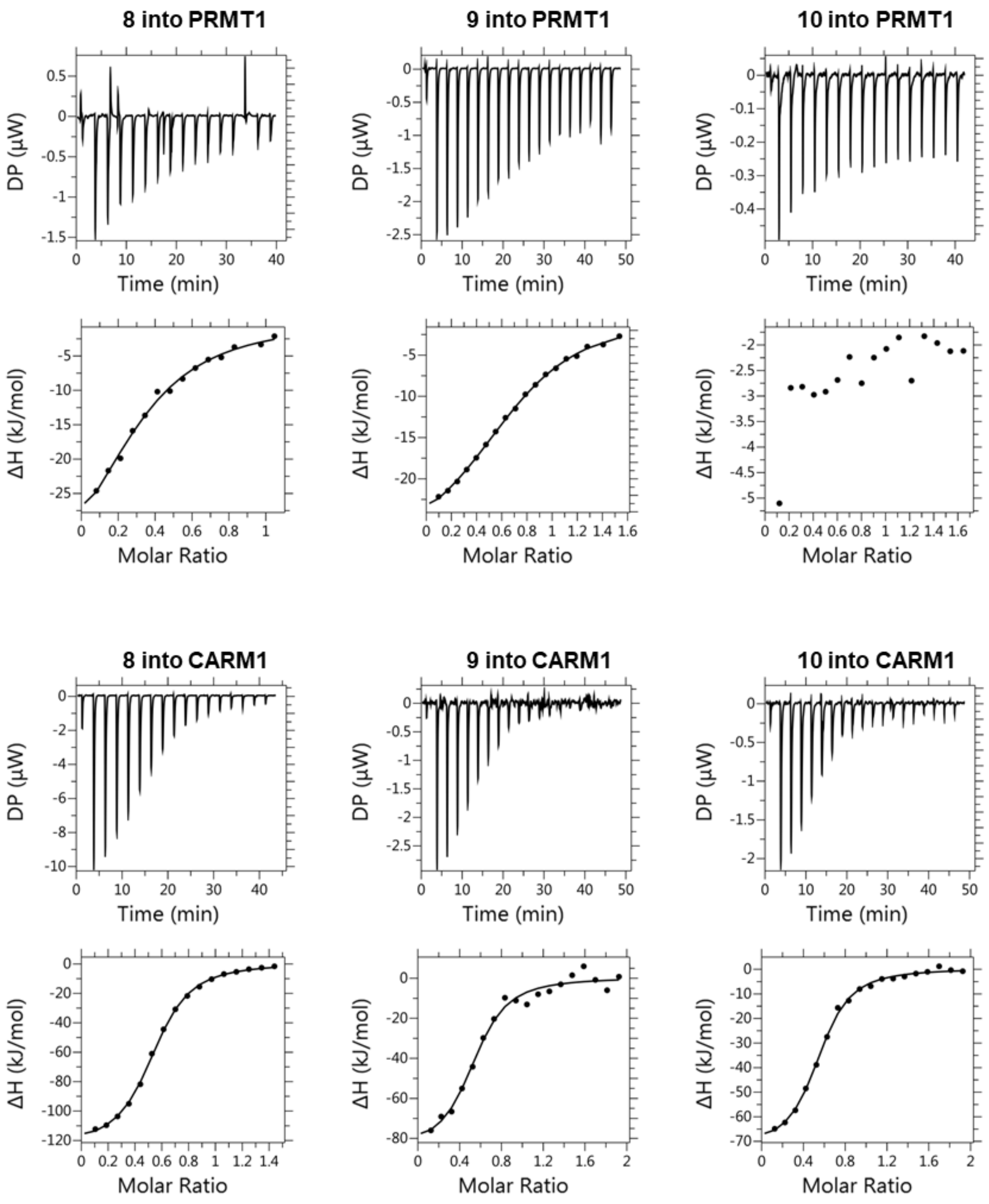

Supplementary Figure S4. ITC thermograms (upper panels) and integrated heat plots (lower panels) for inhibitors 8, 9 and 10.

The inhibitor and PRMT used for each titration are given above the relevant graphs. CARM1 and PRMT1 titrations were conducted at 25°C with PRMT concentrations in the range of 21 – 80 μM and inhibitor concentrations in the range of 210 – 600 μM in order to optimise sigmoidicity.

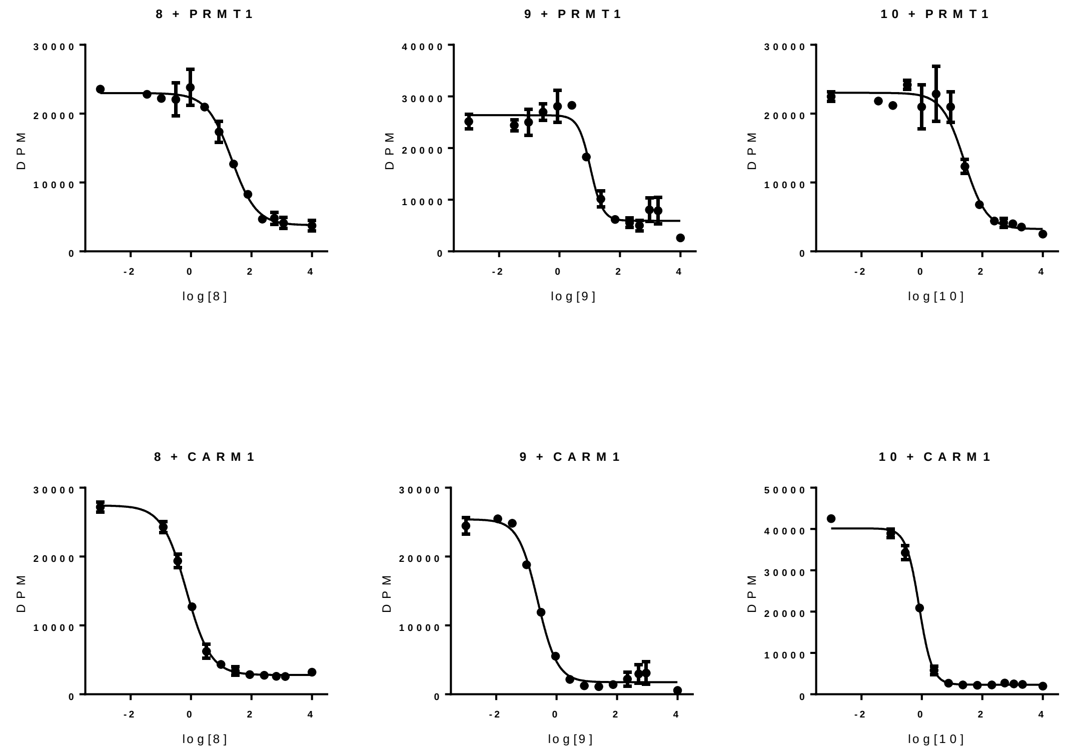

Supplementary Figure S5. Dose-response curves for inhibitors 8, 9 and 10.

IC_{50} curves for inhibitors **8**, **9** and **10** with PRMT1 and CARM1. Error bars indicate the S.D. (n=2) at each inhibitor concentration. DPM is the un-normalised disintegrations per minute, measured by liquid scintillation counting, and is proportional to tritium incorporation into the substrate.

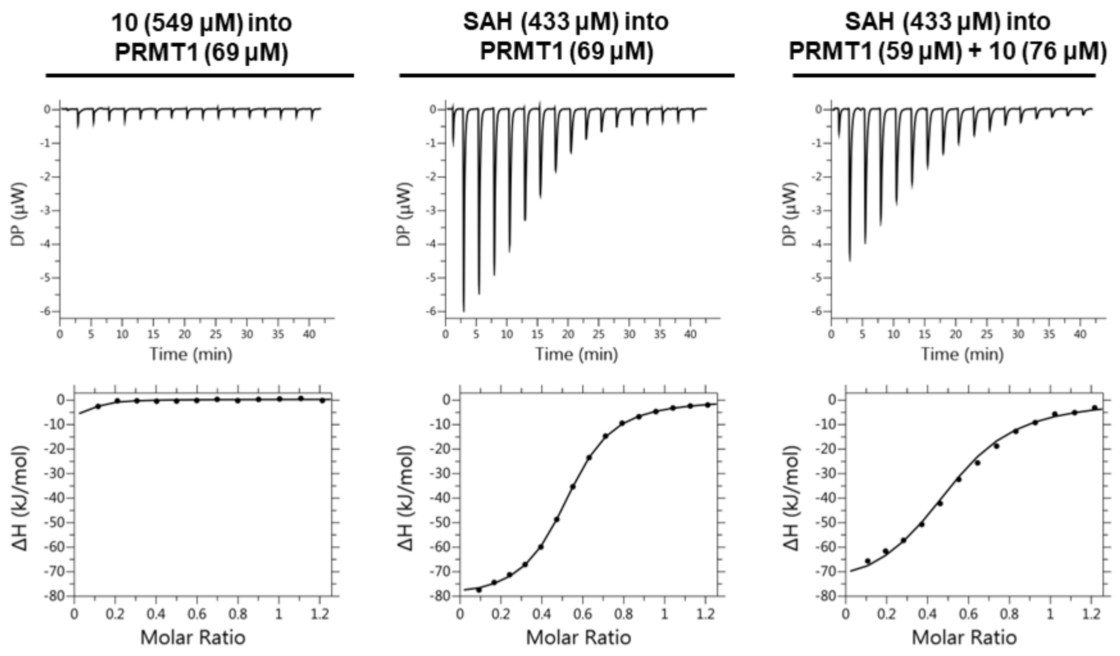

Supplementary Figure S6. ITC experiments with PRMT1 and inhibitor 10 or SAH.
ITC thermograms (upper panels) and integrated heat plots (lower panels) for titrations of SAH into PRMT in the presence and absence of inhibitor **10** (i.e. competition ITC experiment) are shown with the respective titrant(s) indicated above the thermograms.

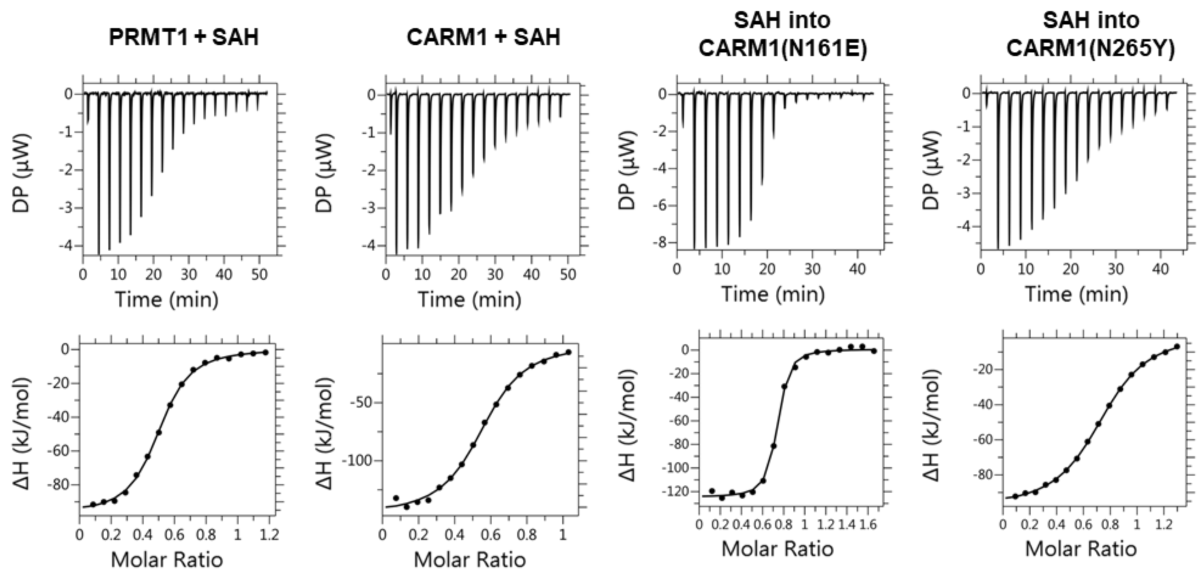

Supplementary Figure S7. ITC data for CARM1 mutants N161E and N265Y and SAH.

ITC thermograms (upper panels) and integrated heat plots (lower panels) for SAH titrations with wild-type PRMT1 and CARM1, and CARM1 mutants N161E and N265Y. The PRMT used for each titration is given above the relevant graph. Titrations were performed at 25°C with 200 µM SAH and 35 µM PRMT1. CARM1 concentrations ranged from 30–35 µM and SAH concentrations from 150 – 264 µM.

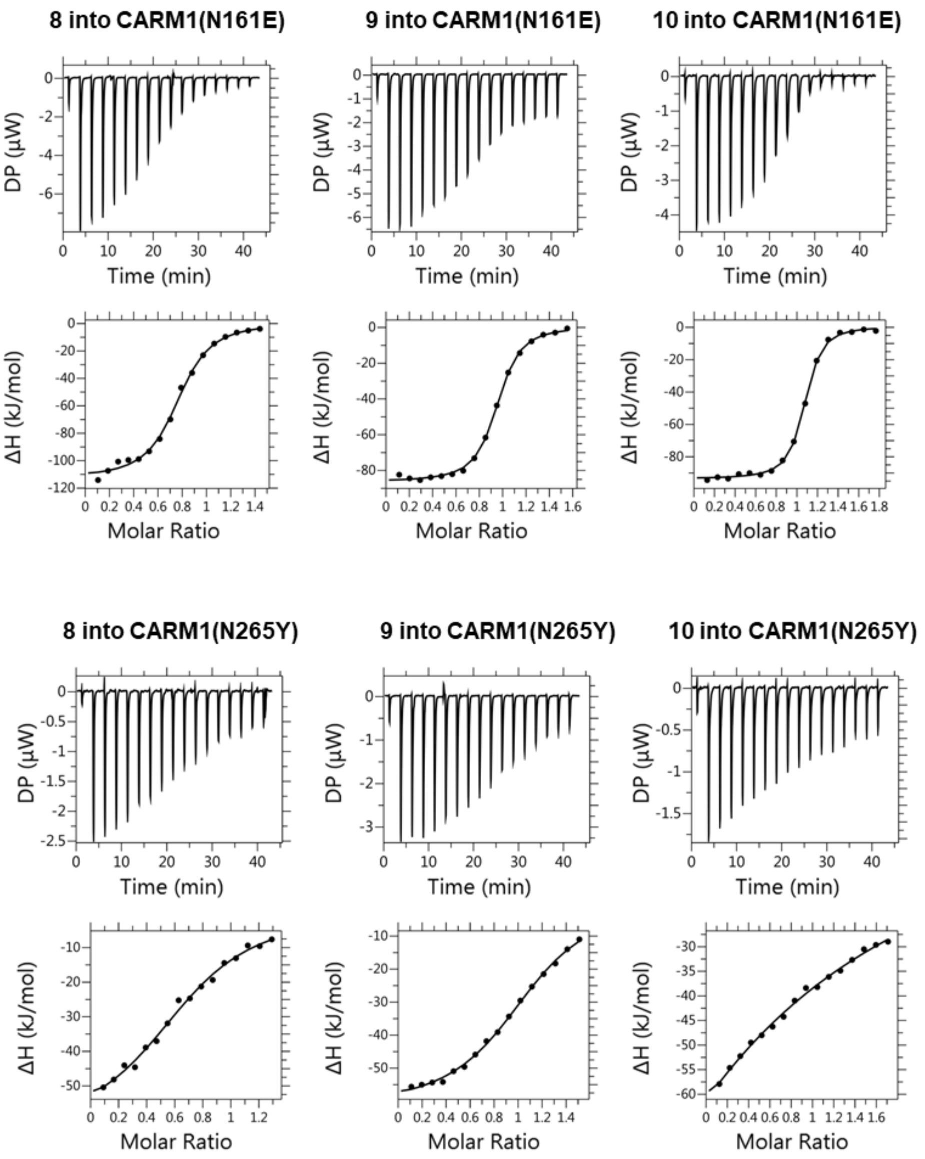

Supplementary Figure S8. ITC thermograms (upper panels) and integrated heat plots (lower panels) for inhibitors 8, 9 and 10 with CARM1 mutants N265Y and N161E.

The inhibitor and CARM1 mutant used for each titration are given above the relevant graphs. Titrations were performed at 25°C with CARM1 concentrations from 30 – 46 µM and inhibitor concentrations from 219 – 320 µM.

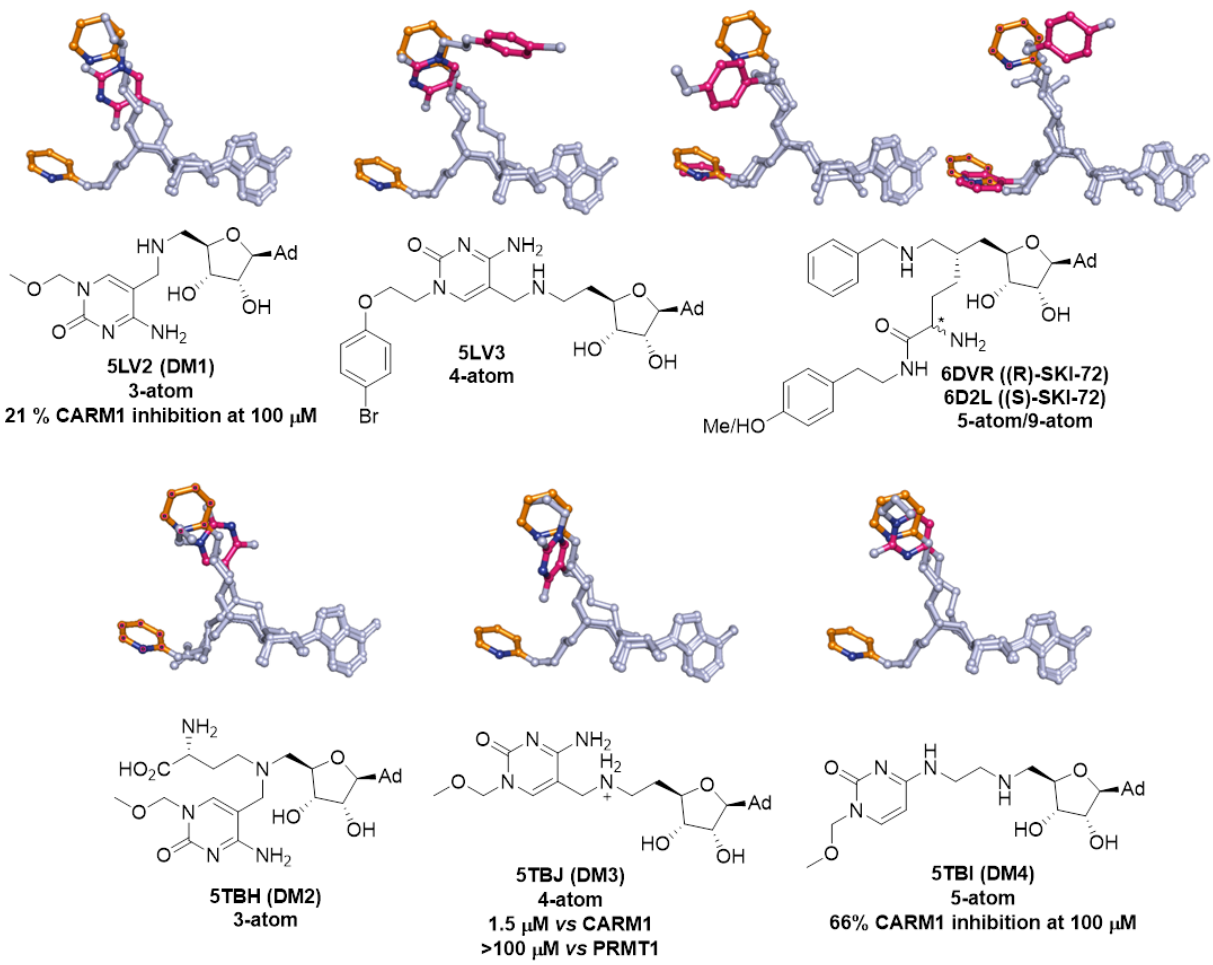

Supplementary Figure S9. Superposition of CARM1-9 complex structure with aromatic bisubstrate inhibitors co-crystallised with CARM1 in the PDB.

Ligands are shown in grey, with aromatic groups highlighted in orange (**9**) and hot pink (superposed inhibitors). Inhibitor structures, respective PDB codes and IC_{50} values (where available) are given underneath the relevant superposition. The number of atoms between the 4’ adenosine carbon and the aromatic group are also indicated. The numbering of compounds DM1-DM4 is used herein and does not correspond to numbering used in the original paper.
